# Phaseolotoxin: Environmental Conditions and Regulatory Mechanisms Involved in Its Synthesis

**DOI:** 10.3390/microorganisms12071300

**Published:** 2024-06-26

**Authors:** Jackeline Lizzeta Arvizu-Gómez, Alejandro Hernández-Morales, Juan Campos-Guillén, Christian González-Reyes, Juan Ramiro Pacheco-Aguilar

**Affiliations:** 1 Secretaría de Investigación y Posgrado, Centro Nayarita de Innovación y Transferencia de Tecnología (CENITT), Universidad Autónoma de Nayarit, Tepic 63000, Mexico; 2 Facultad de Estudios Profesionales Zona Huasteca, Universidad Autónoma de San Luis Potosí, Ciudad Valles 79060, Mexico; 3 Facultad de Química, Universidad Autónoma de Querétaro, Santiago de Querétaro 76010, Mexico; juan.campos@uaq.mx (J.C.-G.); juanramiro29@yahoo.com.mx (J.R.P.-A.); 4 Unidad Académica de Ciencias Químico Biológico y Farmacéuticas, Universidad Autónoma de Nayarit, Tepic 63000, Mexico; christian.gonzalez@uan.edu.mx

**Keywords:** phaseolotoxin, *Pseudomonas syringae* pathovars phaseolotoxin-producers, regulation, Pht cluster, Pbo cluster, thermoregulation

## Abstract

Phaseolotoxin is an antimetabolite toxin produced by diverse pathovars of Pseudomonas syringae which affects various plants, causing diseases of economic importance. Phaseolotoxin contributes to the systemic dissemination of the pathogen in the plant, therefore it is recognized as a major virulence factor. Genetic traits such as the Pht cluster, appear defining to the toxigenic strains phaseolotoxin producers. Extensive research has contributed to our knowledge concerning the regulation of phaseolotoxin revealing a complex regulatory network that involves processes at the transcriptional and posttranscriptional levels, in which specific and global regulators participate. Even more, significant advances in understanding how specific signals, including host metabolites, nutrient sources, and physical parameters such as the temperature, can affect phaseolotoxin production have been made. A general overview of the phaseolotoxin regulation, focusing on the chemical and physical cues, and regulatory pathways involved in the expression of this major virulence factor will be given in the present work.

## 1. Introduction

**Figure 1:**
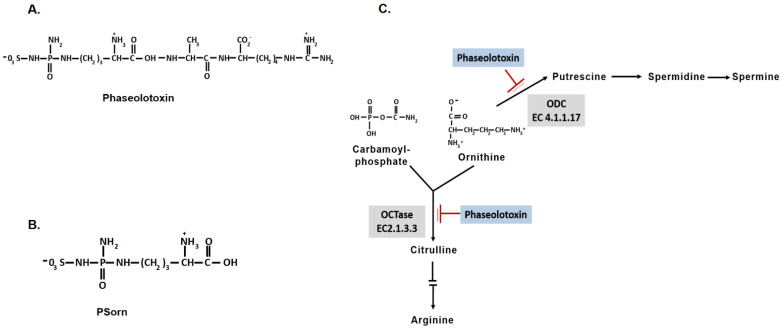
Structure and mechanism of action of phaseolotoxin. (**A**) Chemical structure of phaseolotoxin. N^δ^-(N′-sulphodiaminophosphinyl)-ornithyl-alanyl-homoarginine. (**B**) Structure of octicidine [4]. Phaseolotoxin is cleaved by plant peptidases releasing the alanine and homoarginine residues, which results in octicine (Psorn) formation. (**C**) Targets of action of phaseolotoxin. The red lines indicate the biochemical reactions affected by the action of phaseolotoxin on the catalytic enzymes of these processes.

Phaseolotoxin is an antimetabolite toxin that is chlorosis-inducing and non-host specific, and is produced by *Pseudomonas syringae* pv. *phaseolicola* (syn *P. savastanoi* pv. *phaseolicola*; *P. amygdali* pv. *phaseolicola*) and *P. syringae* pv. *actinidiae*, which cause halo blight on legumes (bean; *Phaseolus vulgaris* L. and mungbean; *Vigna radiata* L.) and bacterial canker on kiwifruit, respectively [1]. Only a strain of *P. syringae* pv. *syringae* (CFBP3388 strain) belonging to the epiphytic microflora from Vetch (*Vicia sativa*) has also been identified as a phaseolotoxin producer [2]. Phaseolotoxin structure was initially elucidated by Mitchell (1976) [3] and revised by Moore et al. (1984) [4]. Phaseolotoxin consists of a sulphodiaminophosphinyl moiety linked to a tripeptide consisting of ornithine, alanine, and homoarginine referred to as [N^δ^-(N′-sulphodiaminophosphinyl)-ornithyl-alanyl-homoarginine] (Figure 1A). Phaseolotoxin competitively inhibits the ornithine carbamoyltransferase enzyme (OCTase; EC2.1.3.3), which converts ornithine and carbamoylphosphate to citrulline in the arginine biosynthesis pathway (Figure 1) [5,6]. Although phaseolotoxin is a reversible inhibitor of OCTase, it is hydrolyzed in planta by peptidases to produce N^δ^-(N′sulphodiaminophosphinyl)-l-ornithine, also called octicidine or Psorn (Figure 1B). Unlike phaseolotoxin, octicidine is an irreversible inhibitor of OCTase and the predominant form of the toxin in infected tissue [7,8]. The inhibition of OCTase causes an accumulation of ornithine and a deficiency in intracellular pools of arginine, leading to chlorosis [9]. Phaseolotoxin inhibits further another enzyme of ornithine metabolism, orithine decarboxylase (ODC; EC 4.1.1.17), which is a key enzyme in the polyamine biosynthetic pathway and the cellular cycle (Figure 1C). Thus, the action of phaseolotoxin results in the inhibition of chlorophyll synthesis and chlorophyll destruction [9,10]. Although phaseolotoxin is not essential for the pathogenicity or the development of diseases in the host plants [11,12], it has been recognized as an important virulence factor, in particular of *P. syringae* pv. *phaseolicola*, because it contributes to the systemic dissemination of bacterial pathogens in their host [13]. Phaseolotoxin synthesis in coordination with the pathogenic status of the bacteria in the plant environment is a crucial task that requires fine and tight control for the success of the infection process. Thus, it is important to elucidate and know the molecular bases and regulatory pathways related to phaseolotoxin synthesis to understand the survival strategies and evolution of phaseolotoxin-producing pathogens.

## 2. Genetic Determinants Related to Phaseolotoxin Production

### 2.1. Pht Cluster (argK-Tox Cluster or Tox-Island)

The toxigenic ability (Tox^+^), or phaseolotoxin production, has been attributed mainly to the carriage of genes belonging to a 30,245 bp chromosomal region called the “Phaseolotoxin (Pht) cluster” (previously referred to as the *argK-tox* cluster or *tox*-island). The presence of insertion sequences and related transposases at both ends of the Pht cluster together with its G+C content of 51.9%, which contrasts with the 58% of the *P. syringae* pv. *phaseolicola* chromosome, have suggested that this entire region has been acquired by horizontal transfer [14,15]. The close phylogeny of some genes of the Pht cluster with those from Gram-positive species, with a similar G+C content, suggests an origin of genes for the biosynthesis of phaseolotoxin from a Gram-positive microorganism [16,17]. However, so far there is not experimental evidence that demonstrates whether the Pht cluster contains all necessary elements for phaseolotoxin production.

**Figure 2:**
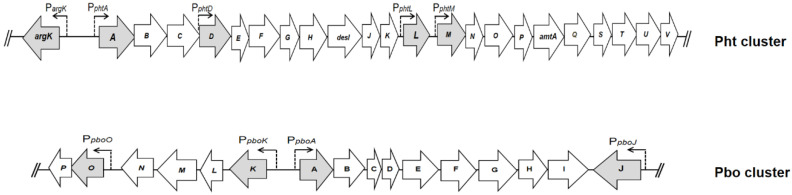
Genomic determinants involved in phaseolotoxin synthesis. Graphic representation of the Pht and Pbo regions. Arrows represents an individual gene, whose direction indicates the direction of transcription. Gray arrows indicate the first gene of each transcriptional unit [14,24].

The Pht cluster (30.25 kb) consists of 27 open reading frames (ORFs), of which three ORFs at the 5′end and regions at the 3′end show homology with insertion sequences or transposases. Between these ends, 23 ORFs were identified, each one preceded by a putative ribosome-binding sequence, which was organized into five transcriptional units: two single-gene units, *argK* and *phtL*, and three operons, a large operon from *phtA* to *phtK*, with an internal promoter driving the expression of *phtD* to *phtK*, and a third operon including genes from *phtM* to *phtV* (Figure 2) [14]. So far, functions have been assigned to only a few of these genes: *argK* plays dual roles for self-defense, with the coding capacity of a phaseolotoxin-insensitive ornithine carbamoyltransferase (ROCT) that provides an alternative arginine source thereby protecting the bacteria from the harmful effects of its own toxin [18,19], and the control of the phaseolotoxin production by converting biosynthetic precursors to nontoxic Cit-Ala-*h*Arg, with tripeptide Orn-Ala-*h*Arg and carbamyl phosphate as substrates [20]; *amtA* encodes an amidinotransferase responsible for homoarginine biosynthesis [21]; and *phtU* encodes an L-amino acid ligase that synthesizes alanyl-homoarginine, part of the phaseolotoxin scaffold [22]. The *desI* gene shows significant homologies to genes coding for fatty acid desaturases, but its function has not yet been determined [23].

The participation of the Pht cluster genes in phaseolotoxin synthesis was identified from studies in the *P. syringae* pv. *phaseolicola* NPS3121 strain, in which, by the generation and analysis of mutants strains in some of these genes, it was demonstrated that they played a role in the production of this phytotoxin, which could be involved at any of the different stages of its production, such as synthesis, transport, and/or regulation. The influence of the Pht cluster genes in phaseolotoxin synthesis has been shown to be variable. Thus, polar and non-polar mutations in most of the Pht cluster genes result in a phenotype non-toxigenic (Tox^−^) or non-phaseolotoxin producer, with exception of the *desI*, *phtO*, and *phtT* genes, whose mutation only results in low phaseolotoxin levels [14]. The complementary analysis of genomic elements related the expression or regulation of the genes of the Pht cluster through the mapping of the site of transcription initiation and upstream sequences analysis of each operon identified for *phtA*, a G nucleotide positioned at 87 bp upstream of the ORF as the transcription start site (+1). Furthermore, the upstream region *phtA* contains well conserved −10 and −35 regions characteristics of a Pribnow-type promotor and A-rich regions that could be implicated in RNA polymerase binding. The divergent promoter of *argK* contains the hexanucleotides 5′-TTGACA-3′ and 5′-TAaAAc-3′, centered at positions −32 and −8, respectively, from the transcriptional start point, which also match the −10 and −35 consensus hexanucleotides of canonical of *E. coli* promoters. The *argK* ORF starts with an ATG codon at positions +125 to +128 from the transcription start point [14,25]. The analysis of the transcription initiation site for *phtD*, *phtL*, and *phtM* identified these as 127, 64, and 73 bp, respectively, upstream from the ORFs. Furthermore, the promoter regions for these genes (*phtD*, *phtM*, and *phtL*) did not show similarity to any of the known σ factors. However, six conserved regions within 60 bp upstream of the site of the transcription initiation of the *phtD* and *phtM* operon were found, suggesting that both are under a common mechanism of transcriptional regulation [14].

The analysis at the nucleotide sequence level of the Pht cluster among strains and phaseolotoxin-producing pathovars has shown, in most cases, minimal or negligible differences among them. The comparison of the sequence of the Pht cluster from the *P. syringae* pv. *phaseolicola* NPS3121 strain with those correspondingly reported for *P. syringae* pv. *phaseolicola* 1448A (PSPPH_4319 to PSPPH_4299), found 35 nucleotide differences; of these, the most significant difference is the presence of a 9 bp sequence in NPS3121 which is missing within locus PSPPH_4306 of 1448A. This introduces a stop codon leading to two ORFs in this locus in NPS3121 instead of one [14]. Likewise, only 11 nucleotide differences were found between the common region containing the Pht-cluster (*argK-tox* cluster) of *P. syringae* pv. *phaseolicola* MAFF302282 and the *P. syringae* pv. *actinidiae* strain KW-11. Furthermore, the comparison of the Pht cluster among diverse strains of pv. *actinidiae* and pv. *phaseolicola* demonstrated that this region is highly conserved among these pathovars [26], even though these two pathogens are phylogenetically separated and could even belong to different species [15,26,27,28,29,30]. The Pht cluster (*argK*-tox cluster or *tox*-island) of pv. *actinidiae* and pv. *phaseolicola* were confirmed to integrate site-specifically into their respective chromosomes at the homologues sites in the same direction [26,31]. Conversely, the analysis of the sequence of the Pht cluster in the *P. syringae* pv. *syringae* CFBP3388 strain indicated that this is only partially conserved; furthermore, it seems to have been inserted in a position different from that in other phaseolotoxin producers. The variation in the conservation of the cluster for the biosynthesis of phaseolotoxin in *P. syringae* pathovars suggests at least two events of horizontal acquisition [26].

### 2.2. Pbo Cluster (Phaseolotoxin Biosynthesis Operon)

A second genomic island in *P. syringae* pv. *phaseolicola* 1448A, called the Pbo cluster (phaseolotoxin biosynthesis operon) (19,985 bp), has been identified as involved in phaseolotoxin synthesis. This region, made up 16 genes, encompassing the PSPPH_4538 to PSPPH_4559 CDSs, is organized in four operons, including three polycistronic and one monocistronic. A large polycistronic operon encompasses nine genes, *pboA-pboI* (PSPPH_4550 to PSPPH_4557 and *pboD*, which has not been annotated). The second transcriptional unit contains four genes *pboK* to *pboN* (PSPPH_4546 to PSPPH_4549), and the *pboO* (PSPPH_4545) and *pboP* (PSPPH_4544) genes that are transcribed together, comprising another polycistronic operon. Finally, the monocistronic operon made up by the *pboJ* (PSPPH_4558) gene (Figure 2) [24]. The organization of the Pbo cluster is conserved with high identity in the NPS3121 strain. By polar mutants assays in different genes of the Pbo cluster, the participation of these genes in the phaseolotoxin synthesis is evident. Mutations within the *pboA* transcriptional unit (*pboA*, *pboC*, *pboE*, and *pboG* genes) and *pboO^−^* mutant resulted in a non-toxigenic (tox-) phenotype, while four mutations (*pboJ*, *pboK*, *pboM*, and *pboL*) exhibited only low levels of toxin production [24].

The Pbo cluster region is flanked by putative transposases and direct repeats and shows a GC content of 48%, contrasting with the 57.8% GC for the 1448A chromosome. Furthermore, the presence of this region in diverse pathovars suggests a possible horizontal transfer origin for the Pbo cluster, similar to those proposed for the Pht cluster. The analysis of the conservation and phylogeny of the Pbo cluster has demonstrated a limited distribution among Pseudomonads, which is not associated with the Pht cluster. Thus, *P. syringae* pv. *actinidiae* bacteria, phaseolotoxin producers and carriers of the Pht cluster do not contain the Pbo cluster. Similarly, diverse *P. syringae* pathovar non-phaseolotoxin producers contain sequences with high identity to the Pbo cluster. Based on the conserved domains and annotation of the individual genes, the Pbo cluster is likely involved in the biosynthesis of a secondary metabolite(s) resulting from the action of non-ribosomal peptide synthetases (NRPs) and polyketide synthetases (PKs), which is postulated to participate in the regulation of phaseolotoxin synthesis [24].

## 3. Environmental Signals

Knowledge about the signals and/or factors that influence the synthesis of phaseolotoxin or on elements related to it, contribute to the understanding of the biology and physiological context related to the virulence of phaseolotoxin-producing *P. syringae* pathovars. This knowledge also provides a perspective on the possible regulatory mechanisms involved in this process. A few works have aimed their research at this topic, and from them, diverse cues and biotic or abiotic factors have been identified to have influence on phaseolotoxin synthesis (Table 1).

### 3.1. Influence of Low Temperatures in Phaseolotoxin Synthesis

The studies in diverse strains and pathovars of *P. syringae*, producers of phaseolotoxin, have highlighted the importance of the temperature, particularly low temperature conditions, as a key environmental factor for the synthesis of this compound. After establishing the relationship between low temperatures and the development of the halo blight disease [39] caused by *P. phaseolicola*, the initial works aimed at the purification and structural elucidation of the phaseolotoxin used cultures conditions at 18 °C from which the purification of this antimetabolite toxin was successfully obtained [3]. Later works demonstrated that, in *P. phaseolicola* cultures grown at temperatures above at 18 °C, the phaseolotoxin content of the medium decreased progressively with an increase in temperature [32]. In cultures of *P. syringae* pv. *phaseolicola* grown at 16 °C, 912 µg of phaseolotoxin/L medium was obtained; at 20.5 °C, 628 µg/L; and at 25 °C, 290 µg/L. No detectable phaseolotoxin was found in cultures grown at 28 °C [32]. Thus, it was demonstrated that phaseolotoxin production by *P. syringae* pv. *phaseolicola* strongly depends on the cultivation temperature, with a high rate of toxin production observed only at temperatures below 20 °C (8 °C to 18 °C) with 18 °C being the optimal temperature for phaseolotoxin production and with little or no detectable toxin being produced at 30 °C [32,40]. Similarly, the conditions used for the initial purification and characterization of phaseolotoxin from the *P. syringae* pv. *actinidiae* Kw11 strain corresponded to those previously reported [3] which used low temperatures (18 °C) for successfully obtaining phaseolotoxin [41,42]. Conversely, the independence of low temperature conditions for phaseolotoxin synthesis has only been observed in the *P. syringae* pv. *syringae* CFBP3388 strain, which produces phaseolotoxin at 28 °C, a non-permissive temperature for other phaseolotoxin producers [26].

Relatively recent studies in the *P. syringae* pv. *phaseolicola* NPS3121 strain focused on the functional evaluation of genomic traits identified as being involved in phaseolotoxin synthesis have demonstrated further the influence of the low temperatures (18 °C) on the expression of phaseolotoxin genes and the influence of this environmental factor in the transcriptional regulation of these genes. The qualitative transcriptomic analysis of the Pht cluster genes by RT (reverse transcriptase)-PCR and Northern blot assays demonstrated that these genes are transcribed at high levels at 18 °C and that most show some basal level of expression at 28 °C, with the exception of the *phtL* gene whose expression was detected at temperatures permissive, 18 °C, and nonpermissive, 28 °C, for phaseolotoxin production [14]. Furthermore, the transcriptional fusions in the *cis* and *trans* analysis of promoters of the Pht cluster genes showed a differential expression pattern dependent on temperature, with higher expression levels at 18 °C in relation to 28 °C, with the exception of the *argK* promoter, which showed similar levels of expression at both temperatures. These results demonstrated the regulation of the expression of Pht cluster genes mediated by low temperatures (18 °C) (thermoregulation) [14]. Thus, the requirement of low temperature conditions for the production of phaseolotoxin is related to the fact that this environmental factor is key in the regulation of the expression of genes involved in the synthesis of this compound.

Similarly, the thermoregulation of most of the Pbo cluster genes was also demonstrated [24]. By RT-PCR analyses, it was observed that the genes *pboO*, *pboN*, *pboM*, *pboL*, *pboK*, *pboA*, *pboC*, *pboE*, *pboF*, and *pboG*, belonging to the *pboO-pboP*, *pboK-pboN*, and *pboA-pboI* operons, are transcribed at high levels at 18 °C but with undetectable or basal levels of expression at 28 °C. Only the monocistronic transcriptional unit *pboJ* was constitutively expressed at both temperatures (28 °C and 18 °C) [24,33,43]. Although the expression pattern of the Pbo cluster genes as a function of temperature has been validated for the majority of these genes by various studies, different expression patterns have been reported for the *pboM* gene (PSPPH_4547), in which a differential expression at low temperatures (18 °C) and even similar expression at both temperatures (28 °C and 18 °C) has been observed [24,33]. However, this fact could be related to the qualitative techniques used in these studies.

Because low temperatures (18 °C) are a key factor in the pathogenicity and virulence (e.g., phaseolotoxin synthesis) in *P. syringae*, global analyses on the physiology of the bacteria under this condition have been carried out, delving into the behavior and molecular-cellular events related to this environmental factor. By microarray assays, the transcriptional profile at low temperatures (18 °C) of the *P. syringae* pv. *phaseolicola* NPS3121 bacterium was described. The transcriptional profile obtained at 18 °C makes sense with a response to oxidative stress [33]. Therefore, these analyses suggest that low temperatures (18 °C) initially induce oxidative stress in the *P. syringae* pv. *phaseolicola* cells, which in turn, give rise to the expression profile of the oxidative stress response obtained. This transcriptional profile highlights the expression of the Pht and Pbo cluster phaseolotoxin genes suggesting a link between oxidative stress and phaseolotoxin synthesis [33]. The relation between these two events has already been demonstrated (see below).

### 3.2. Culture Conditions. Carbon Source, Incubation Time, and Medium Composition

Early works aimed at time-course studies of phaseolotoxin production date back to 1976. Four isolates identified and named as *Pseudomonas phaseolicola* were evaluated throughout their growth in in vitro cultures for phaseolotoxin production. The time-course studies showed that phaseolotoxin production is performed during the first 24 h of bacterial cultures showing only slight increases in the synthesis of this antimetabolite between the 48 and 72 h of incubation. No increase in phaseolotoxin production was observed afterward. Complementary studies of these *P. phaseolicola* isolates during their interaction with host plants demonstrated that the phaseolotoxin production was carried out from 2 to 3 days post-inoculation. However, the time necessary for the synthesis of this antimetabolite depended on the cultivar of the host plant with which the bacteria interacted [34].

Additionally, the influence of carbon sources in phaseolotoxin synthesis has also been established, in which their effect appears to be directly related to their potential for biomass formation [35]. While phaseolotoxin production by *P. phaseolicola* was observed to be independent of the carbon or energy source, its production is favored with a C source, which leads to a high growth rate. Depending on the nature of the C and energy sources, *P. phaseolicola* transfers 2 to 6% into biosynthetic products, including phaseolotoxin. But with sucrose as a substrate, the overall phaseolotoxin proportion of the product mixture amounts to up to 6.5%, while with ribose as a substrate it is only 1.0%. The sucrose constitutes the natural C-source of this phytopathogenic bacterium in the bean host plant, which is congruent with the fact that this C-source (sucrose) is one of the best for the biosynthesis of biomass and phaseolotoxin [35].

Recent efforts in these topics focused on evaluating the influence of the medium composition in the expression of phaseolotoxin genes, and were performed in the *P. syringae* pv. *actinidiae* biovar 6 (Psa6 MAFF 212134) strain, which is characterized by being a producer of two phytotoxins: phaseolotoxin and coronatine [36]. By RNA sequencing (RNAseq) analysis evaluating the expression profile of the *P. syringae* pv. *actinidiae* biovar 6 strain grown over 3 h in HS or HSC medium (both inducers of the coronatine synthesis) at 18 °C and 27 °C, it was observed that a non-significant change in the expression of phytotoxin genes occurred under the conditions used. This event indicated that the expression of genes of phytotoxins, particularly phaseolotoxin genes, is a function of time and that the incubation time (3 h) used in these assays was limiting to producing these compounds in the Psa6 strain [36]. Therefore, complementary studies by RT-qPCR assays evaluating the temporal changes (0, 3, 6, and 12 h) in the expression of virulence genes, including the phaseolotoxin genes *argK* (encoding OCTase, phaseolotoxin-resistant) and *argD* (encoding ornithine aminotransferase), under various culture conditions (HS, HSC, LB media, and *hrp*-inducing medium at 18 °C or 27 °C) were performed. The assays showed the induction of the *argK* and *argD* genes in HS and HSC media while no change in the expression of these genes was observed in *hrp*-inducing medium, which is known to induce *hrp*-dependent effector genes [36].

The expression analysis in the time-course analysis showed a differential expression in phaseolotoxin genes, *argK* being part of the early-inducible genes, which were induced after 3 h of incubation and suppressed at 6 h and 12 h, while the *argD* gene was induced after 6 h of incubation. The latter was expressed longer than the *argK* gene. Surprisingly, the incubation temperature (18 °C or 27 °C) did not result in significant changes in gene expression despite the fact that this environmental factor plays a key role in the synthesis of phaseolotoxin in other producer strains (mentioned above). This finding demonstrated that the culture medium composition and incubation time can have a stronger effect than incubation temperature in some strains, such as *P. syringae* pv. *actinidiae* biovar 6 [36]. From this transcriptomic analysis, it is known that, depending on the type of Pseuodomonas (pathovars and strains), phaseolotoxin production is affected by various environmental conditions [36].

### 3.3. Plant Metabolites

During interaction with the host plant, and under favorable environmental conditions, the phytopathogenic bacteria express their arsenal or machinery of pathogenicity and virulence, which allow them to multiply and cause disease on plants. Many of these genic determinants are induced only in planta or in the presence of host components, suggesting that gene expression is regulated by signals that bacteria receive from plant tissue.

By microarray analysis in *P. savastanoi* pv. *phaseolicola* NPS3121, it was demonstrated that extracts or tissue of the *Phaseolus vulgaris* L. host plant have an influence on the expression of phaseolotoxin genes (Pht and Pbo cluster), which is variable in the function of the plant’s tissue extract (bean leaf extract, pod extract, or apoplastic fluid). A positive or negative influence on the expression of these genes by the plant extracts/tissue was independent of low temperatures (18 °C). Bean leaf extracts and apoplastic fluid enhanced the expression at 18 °C of the *phtB* and *desI* genes, belonging to the *phtA* and/or *phtD* operon, (Pht cluster), respectively. Likewise, increased levels of the transcription of the *phtM*, *phtO*, *amtA*, *phtQ*, *phtS*, *phtT*, and *phtU* genes of the *phtM* operon and the *phtL* gene were obtained in presence of these plant extracts at 18 °C [37]. Conversely, the *argK* gene and Pbo cluster genes did not show changes in their expression in the presence of bean leaf extracts or apoplastic fluid at 18 °C. On the other hand, assays with pod extracts showed the ability of these to decrease the expression of Pbo cluster genes, particularly the *pboG* gene (PSPPH_4555) belonging to the largest transcriptional unit *pboA*, and the *pboP* (PSPPH_4544) gene of the *pboO-pboP* operon. Regarding the Pht cluster genes, only the expression of the *argK* gene was negatively affected by the presence of pod extracts while the rest of the genes did not have changes in their expression at 18 °C under the presence of pod extracts [37]. Thus, it is now known that, for the optimum expression of some phaseolotoxin genes, specific plant components of leaf and apoplast are required in addition to the low temperature conditions (18 °C) during the interaction between the host plant and bacteria.

The physiological context of *P. savastanoi* pv. *phaseolicola* cells under conditions of bean leaf extracts and apoplastic fluid corresponds mostly to non-limiting iron conditions. The Fur global regulator protein involved in iron homeostasis is induced in the presence of plant extracts (leaf and apoplast) while uptake and metabolism iron genes are decreased [37]. This could suggest a link between iron metabolism and phaseolotoxin synthesis.

### 3.4. Oxidative Stress

The influence, previously suggested, of oxidative stress on phaseolotoxin synthesis genes [33] was finally demonstrated. Oxidative stress has a profound effect on the expression of phaseolotoxin genes, particularly on the Pht cluster genes, which produce a differential expression pattern dependent on reactive oxygen species (ROS) and the concentration of these in the cells [38]. It was observed that the expression dependent on a low temperature (18 °C) (thermoregulation) of some or all genes of the Pht cluster is lost in the presence of H_2_O_2_. This alleviation of thermoregulation further depends on the concentration of the oxidizing agent (H_2_O_2_). Thus, 1 mM H_2_O_2_ concentrations lead to the expression at 28 °C of all gene representatives of each transcriptional unit that makes the Pht cluster, while 2.5 mM H_2_O_2_ concentrations influenced only the expression of the *phtD* and *phtL* operons at 28 °C [38].

On the other hand, the superoxide ion (O_2_^−^) exerted both positive and negative effects on the expression of Pht cluster genes at 28 °C, whose expression pattern was similar among the concentrations of the oxidizing agent, but with these effects increased as a function of the concentration. In the presence of the superoxide ion (O_2_^−^)-generating compound (Paraquat), the expression of *argK* and *amtA* genes was induced at 28 °C, while the basal expression of *desI* and *phtL* expression at 28 °C was decreased in the presence of the superoxide ion ( O_2_^−^). *phtA* was not affected in either concentration of the superoxide ion ( O_2_^−^)-generating compound [38]. Thus far, the influence of ROS or oxidative stress on the expression of the Pbo cluster genes has not been evaluated.

On the basis of these results, it is now established that the expression of phaseolotoxin genes is part of the oxidative stress response in *P. syringae* pv. *phaseolicola* and supports the hypothesis that oxidative stress is part of the signal transduction pathway related to the expression of phaseolotoxin genes. Additionally, it was suggested that regulatory mechanisms of the oxidative stress response might be involved in the regulation of phaseolotoxin genes. The OxyR and SoxRS regulons have been identified as the main regulatory pathways for the response to oxidative stress in bacteria, which respond in particular to specific ROS, OxyR responding to H_2_O_2_ and SoxRS to O_2_^−^ ion, leading to the generation of an expression pattern specific for each regulon [44,45,46]. The existence of proteins homologous to the global regulator OxyR and its functional conservation in various bacterial groups, including *Pseudomonas* sp., has been established. In contrast, the function of the SoxRS regulon, involved in the stress response by (O_2_^−^), appears to be moderately conserved among bacterial groups, where in Pseudomonas sp., of the gammaproteobacteria, SoxRS does not regulate a O_2_^−^ stress response but seems to be involved in the expression of efflux pumps [44]. The genome of the *P. savastanoi* pv. *phaseolicola* 1448A strain does not contain a SoxR-encoding gene [47].

### 3.5. Phaseolotoxin Precursors

**Figure 3:**
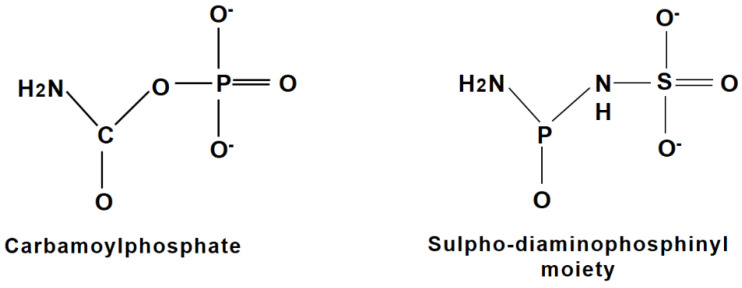
Chemical structure of phaseolotoxin intermediates and similar molecules. (**Left**): Chemical structure of carbamoylphosphate. (**Right**): Structure of the inorganic moiety of phaseolotoxin (sulpho-diaminophosphinyl). The figures have been adapted from [14,49].

Studies using molecules with a chemical structure similar to those of precursors of phaseolotoxin have demonstrated the induction of the expression of some genes of the Pht cluster dependent on these precursors. Analysis in an *argF*- mutant strain, encoding the phaseolotoxin-sensitive OCTase (SOCT) of *P. syringae* pv. *phaseolicola* NPS3121, showed the de-repression at 28 °C of the *argK* gene and the production of the phaseolotoxin-resistant OCTase (ROCT) under this condition, related to non-permissive phaseolotoxin conditions. The evaluation of the influence of the carbamoyl phosphate, whose chemical structure strongly resembles the inorganic moiety of phaseolotoxin, the (N^δ^-(N′sulphodiaminophophynil) group (Figure 3), demonstrated the capacity of this molecule to induce the expression of the *argK* gene at 28 °C. Under these conditions, phaseolotoxin production was not detected, indicating that carbamoylphophate has an effect only on the *argK* gene but not on genes of phaseolotoxin synthesis [19]. Furthermore, the participation of the global arginine regulator ArgR on the expression of the *argK* gene and in phaseolotoxin synthesis was discarded [48]. These results indicated that the *argK* gene is subject to induction and it is not directly regulated by temperature but coordination with phaseolotoxin synthesis is mediated through the synthesis of the inducer which occurs at low temperatures (18 °C) [19].

## 4. Phaseolotoxin Regulation

Genomic studies in diverse phytopathogenic bacteria have demonstrated common characteristics of gene organization in determinants related to the synthesis of phytotoxins. Both structural genes and regulatory proteins involved in the synthesis of these compounds are generally grouped together in a particular chromosomal region [50]. However, for phaseolotoxin, the bioinformatic analysis for each of the predicted ORFs of the Pht cluster and those of the PboO cluster, in a search for DNA-binding motifs, did not identify the presence of these motifs in any of the genes that make up both clusters [14,24,51]. Although, a regulatory function for a few genes of the Pht cluster has been suggested based on their influence on the expression of phaseolotoxin genes [14].

## 5. Pht Cluster-Encoded Regulators

### 5.1. Regulation Mediated by PhtL

By microarray analyses and RT-PCR assays in a *Pseudomonas syringae* pv. *phaseolicola* NPS3121 *phtL*- mutant strain, it was demonstrated that the product of the *phtL* gene positively influences the expression of the genes of the *phtM-phtV* operon at 18 °C, since low transcript levels for these genes are obtained under the loss of function of the PhtL protein [14,52]. Likewise, a feedback regulation by PhtL at 18 °C and 28 °C has been demonstrated, in which the PhtL protein has an influence, in some way unknown, on the expression of its own gene at both temperatures [14]. The influence of PhtL on the Pht cluster genes is selective for these transcriptional units (*phtL* and *phtM*), while in three others (*argK*, *phtA*, and *phtD*), no change in their expression is observed with a lack of *phtL* at 28 °C or 18 °C [14].

Similarly, PhtL has specific influence on the expression of genes that make up the Pbo cluster under low temperature conditions (18 °C). Experimental evidence demonstrates that PhtL favors the expression of the *pboK* gen (PSPPH_4549) at 18 °C [52]. Since this gene is part of the *pboK-pboN* operon [24], it could be considered to have a similar effect to that of the PhtL protein on the rest of the genes of this transcriptional unit. However, no influence of the *phtL* gene on the expression of *pboA* (PSPPH_4550) and *pboG* (PSPPH_4555) genes belonging to the larger transcriptional unit *pboA-pboI* and those of the *pboO-pboP* operon at 18 °C has been determined, since similar transcript levels for these genes are observed in both wild-type (wt) and *phtL*- mutant strains at 18 °C [52]. The influence of PhtL on the expression of the *pboJ* gene has not been evaluated. Similarly, the participation of PhtL on the expression of Pbo cluster genes at 28 °C is still unknown. Based on this, it is important to highlight the fact that the product of the *phtL* gene appears to affect the expression of the Pbo cluster genes whose influence in phaseolotoxin synthesis is not essential (e.g., *pboK* operon), since the loss of these genes only resulted in low phaseolotoxin levels (mentioned above) [24].

Global analyses by microarray assays in *Pseudomonas syringae* pv. *phaseolicola* NPS3121 on the function of the PhtL protein demonstrated the relation of this protein to other diverse cellular processes, with those related to iron metabolism being the most influenced by the PhtL protein [52]. The molecular and biochemical behavior of the cultures of the *Pseudomonas syringae* pv. *phaseolicola* NPS3121 *phtL*- mutant strain simulate that of the wild-type strain cultures grown under conditions of iron sufficiency. RT-PCR assays demonstrated that PhtL negatively regulates the Fur protein, the main global regulator repressor of the genes related to iron metabolism. After the release of the repression exerted by Fur on iron response genes by the PhtL protein, the expression of genes of siderophores synthesis, expulsion pumps, and regulatory proteins (e.g., *pvdS* gene) related mainly to the iron uptake is increased [52]. These findings again suggest a relation between iron metabolism and the expression of phaseolotoxin genes. This is based on the fact that the *phtL* gene product is involved in both cellular processes. Thus, iron presence conditions might influence the expression of phaseolotoxin genes and the production of this antimetabolite toxin. More experimental work is still necessary to demonstrate this. Iron is an essential element required for the growth of nearly all living microorganisms due to its influence on diverse cellular processes. The influence of this element in the regulation of the biosynthesis of various secondary metabolites in bacteria, such as phytotoxins, has been documented. Studies have shown that iron exerts a positive effect on the production of the phytotoxins syringomicin and syringotoxin by *P. syringae* pv. *syringae* and in coronatine synthesis by *P. syringae* pv. *tomato* [50,53,54,55,56]. An increased expression of genes related to the synthesis of these compounds (e.g., *syrB* [syringomycin], *cmaD*, *cmaE*, *cmaA*, *cmaB cmaC*, *cmaT*, *cmaU*, *cfl*, and *cfa1-9* [coronatine]) has been observed in the presence of iron [55,56]. Thus, a similar effect on phaseolotoxin synthesis and on the regulation of related genes by the iron could be carried out.

On the other hand, the previous results of the transcriptional analysis of *P. savastanoi* pv. *phaseolicola* in the presence of bean leaf extracts and apoplast fluid at 18 °C (mentioned above), whose physiological context is like those of the *phtL^−^* mutant strain grown at 18 °C, which simulate non-limiting iron conditions [37,52], suggest that regulation mediated by the PhtL protein on iron metabolism regulators (e.g., Fur protein) is dependent on molecules or metabolites of the host plant. The latter is because the repression of the Fur protein exerted by PhtL protein appears to be abolished by the presence of plant extracts, which induce the *phtL* gene just like the *fur* gene [37]. A similar phenomenon might be occurring in the influence of PhtL on the expression of the *phtM* operon under plant extracts conditions. However, so far, the expression pattern under these conditions is congruent with the positive regulator function of PhtL on the expression of the *phtM* operon [37]. PhtL, the product of *phtL*, is a bidomain enzyme which shows distant similarity to pyruvate phosphate dikinase (PPDK) and phosphoenolpyruvate synthase (PS), which are postulated to be related to the N-P bond formation of phaseolotoxin and involved in the biosynthetic steps of Psorn, with _L_-Orn as a close precursor [20].

### 5.2. Regulation by PhtABC

The participation of the *phtABC* genes in the expression of the genes of the Pht cluster has also been demonstrated. By assays in a *phtA*- polar mutant whose behavior was demonstrated to be exclusively related to the role of the genes *phtA*, *phtB*, and/or *phtC*, changes were observed in the expression pattern of Pht cluster genes at 28 °C and 18 °C [57]. Transcriptional fusions assays demonstrated a feedback regulation by PhtABC on the expression of their own genes. PhtABC participates in repressing its own transcription at both temperatures (18 °C and 28 °C) but with a greater repression at 28 °C. Likewise, the activity of the *phtABC* genes exerts a negative effect on the genes of the *phtD-phtK* operon, particularly at 28 °C. With a lack of PhtABC, the thermoregulation mediated by low temperatures (18 °C) for the expression of the *phtD-phtK* operon is lost, showing in the *phtA*- polar mutant background higher transcript signals or expression of these genes at 28 °C (non-permissive expression temperature) in relation to the wt strain, which shows the negative influence of PhtABC on the *phtD* operon at 28 °C (Aguilera et al., 2017) [57]. Conversely, at 18 °C, the PhtABC proteins have a positive effect on the expression of the *phtD-phtK* genes. The positive effect is not so marked, because only a slight decrease in the transcript levels of genes of the *phtD* operon and diminished expression levels of transcriptional fusions of the *phtD* promoter are obtained at 18 °C in the *phtA*- polar mutant background [57].

Additionally, PhtABC has a positive influence on the expression of the *phtL* gene at both 18 °C and 28 °C, which appears to also be partial. This is on the basis that only a slight decrease in the transcript levels of *phtL* gene is obtained in the *phtA*- polar mutant background at both temperatures. PhtABC exerts further a positive effect on the expression of the *phtM-phtV* operon at 18 °C, which appears to be crucial, as with a lack of PhtABC the expression of the *phtM-phtV* operon at 18 °C is almost completely abolished. This is despite the fact that *phtL* gene expression, which influences the *phtM* operon expression at 18 °C (mentioned above), is still carried out at slightly lower levels relative to the wt strain [57]. This indicates that the regulation of the *phtM* operon mediated by PhtABC proteins could be part of an independent regulatory circuit to that of PhtL.

On the other hand, the *phtABC* gene products have also shown participation in *argK* gene expression by negative control [58]. The thermoregulation by low temperatures (18 °C) of the *argK* gene was abolished in a *phtA*- polar mutant strain, which showed similar transcript levels for this gene at both 18 °C and 28 °C. In the wt strain, the expression of this gene is mainly at 18 °C. An increase in the expression of the *argK* gene and higher OCTase activity at 28 °C were observed when the PhtABC products were absent [58]. By Northern blot assays and transcriptional fusion analyses in *P. syringae* pv. *phaseolicola* strains carrying plasmids containing the *argK* ORF plus upstream divergent promoters (*argK* and *phtA* promoters) and *phtA*, *phtB*, and *phtC* coding regions, it was demonstrated that the repression of *argK* at 28 °C is dependent on *phtABC* gene products [58].

Previous studies have already suggested a regulation under negative control for the *argK* gene at 28 °C by a repressor protein, still unknown, which is postulated to be able to bind to specific DNA motifs present in the promoter region *argK*, called the TRR region (Temperature Thermoregulation) (see below). Additionally, carbamoylphosphate has been demonstrated to induce *argK* expression at 28 °C, bypassing the temperature control. This experimental evidence suggests the participation of an inducer molecule, still unknown, for the efficient expression or transcription of the *argK* gene under phaseolotoxin permissive conditions (18 °C). However, the repressor activity of PhtABC on the *argK* expression at 28 °C is independent of the carbamoylphosphate activity, indicating that both mechanisms belong to different regulatory circuitry [58]. Because in silico analyses of the *phtABC* genes do not show correlation or similarity with DNA-binding proteins, but rather to enzymes related to antibiotic synthesis (e.g., Sulfotransferase, cyth-like phosphatase, and a peptidyl-trna hydrolase, respectively), it has been suggested that the PhtABC products participate in the synthesis of a precursor molecule of phaseolotoxin (putatively sulphodiaminophophynyl moiety), which acts as a corepressor activating an unknown repressor protein which inhibits *argK* expression at 28 °C [57,58]. This unknown repressor protein appears to be a global regulator since it is present in *Escherichia coli* (*E. coli*) and in different *P. syringae* pathovars [58].

## 6. Regulatory Mechanisms Independent of the Pht and Pbo Clusters

### 6.1. Thermoregulatory Region (TRR) Regulation of Phaseolotoxin by Negative Control

Simultaneously with the first studies focused on identifying genetic determinants related to the synthesis of phaseolotoxin, various efforts aimed at elucidating the regulatory mechanisms involved in the process began to be carried out. Initial studies on this topic established that the thermoregulation of phaseolotoxin (18 °C) appears to be negatively regulated, and that after the release of this repression, the production of phaseolotoxin is carried out [18]. The fact that the toxin is produced at 18–20 °C but not at 28 °C suggests that toxin production is inhibited at higher temperatures by regulatory mechanisms involving a repressor. Some studies have demonstrated the constitutive expression of some genes of the Pht cluster in different genetic backgrounds. Thus, minimal clones containing the *argK* gene from *P. syringae* pv. *phaseolicola* expressed constitutively when transferred to *E. coli*, in contrast to the regulated expression observed in the *P. syringae* pv. *phaseolicola* strains. This finding suggests that a repressor molecule normally present in *P. syringae* pv. *phaseolicola* is not present in the *E. coli* background, indicating that the gene is regulated under negative control [18]. The co-ordinate regulation by the temperature of phaseolotoxin resistant-OCTase production (*argK* gene) with the phaseolotoxin is suggested as a similar regulatory mechanism for the rest of the genes. Furthermore, multiple copies of a DNA fragment (485 bp) containing a defined sequence, called a thermoregulatory region (TRR), which contains motifs characteristic of DNA-binding sites, overrides thermoregulation when it is introduced in *P. syringae* pv. *Phaseolicola*, leading to phaseolotoxin production at both 28 °C and 18 °C. This suggested the TRR’s ability to titrate to any repressor molecule at 28 °C. By mobility shift DNA-binding assays, it was demonstrated that extracts of *P. syringae* pv. *phaseolicola* grown at 28 °C contain protein(s) that bind to the TRR in a sequence-specific manner. The amount of this DNA-specific protein is much lower in extracts of the strain grown at 18 °C. This suggests that the protein is involved in the thermoregulation of phaseolotoxin and is titrated by TRR. The TRR sequence has also been identified in *argK* [59]. The analysis of this region indicated that this site contains two repeats of a core motif G/C AAAG (CTTT C/G on the complementary strand) separated by a 5 bp spacer [60]. A DNA sequence search of potential binding sites for regulatory proteins within the TRR showed the presence of OmpR-binding sites and sequences homologous to the IHF-binding site [59]. However, a recent study suggests that the TRR sequence is implicated in the synthesis of the sRNA *rsmY* (see below) [61].

### 6.2. Regulation Mediated by the IHF Protein

Integration host factor (IHF) is a small basic DNA-binding protein conserved in Gram-negative bacteria belonging to the so-called nucleoid associated proteins (NAP’s). IHF is a heterodimer consisting of the closely related proteins IhfA and IhfB, which are encoded by the *ihfA* (*himA*) and *ihfB* (*himD*) genes, respectively [62,63]. Its architectural role in DNA organization and the control of DNA transactions such as transcription, site-specific recombination and transposition have been documented [64,65]. Upon binding, IHF affects the local DNA structure by inducing a U-turn in the DNA, leading to a 160° bend in the DNA [66,67]. This capacity to change the local DNA trajectory underpins the assembly of various higher-order nucleoprotein structures and facilitates long-range interactions underlying the effect of IHF, particularly on gene transcription.

The participation of IHF in the regulation of phaseolotoxin genes so far has been only demonstrated on the the *phtD-phtK* operon of the Pht cluster by electrophoretic mobility shift assays and transcriptional fusion analysis [51]. IHF binds to the promoter region of *phtD* within a 104 bp delimited region spanning the −111 to −8 positions relative to the transcription initiation site. According to the in silico analyses, this 104 bp region contains a binding-site for the IHF protein in positions −64 to −44 (TTTTATTTTTCAGATAAATT), which shares 83% identity with the specific consensus sequence WCARNWNNTTR (where W represents A or T; N is A, T, C, or G; and R represents A or G) widely reported for the IHF protein [51,68]. The implication of both the dT-dA-rich upstream region as well as some bases (C, R and TTR) of the binding-site IHF in the *phtD* promoter, on the binding ability of the IHF protein to this region has been demonstrated. Likewise, implications of specific DNA structures for binding IHF to this 104 bp region of the *phtD* operon have been suggested [51]. The action of the IHF protein on the expression of the *phtD* operon is by negative control. However, because the effect mediated by IHF does not lead to a complete repression of the *phtD* operon but only to a decrease in its expression, it has been suggested that the concerted participation of IHF together with other regulatory proteins still unknown is involved in the negative regulation of *phtD* [51]. In general, the IHF protein functions as an accessory factor in a wide variety of processes including transcription. In many of these processes IHF acts as an architectural element which helps the formation of nucleoprotein complexes by bending the DNA at specific sites. IHF works in conjunction with other transcription factors or even with other NAPs to tune gene expression [65,69].

On the other hand, as mentioned above, a similar regulation mechanism has been proposed for the *phtD* and *phtM* operons based on the presence of six conserved regions in the promoters of both genes, one of these conserved regions corresponds to the TTTCAGAT sequence, which is part of the binding site IHF identified in the *phtD* promoter region [14,51]. However, despite the presence of this conserved sequence among both *phtD* and *phtM* promoters, the IHF protein does not have the ability to bind to the *phtM* operon promoter region (−131 to +168 positions) [70].

This, demonstrates again the diversity and/or complexity of regulatory mechanisms involved in the expression of phaseolotoxin genes, in particular of those that make up the Pht cluster, in which specific regulatory proteins acting in conjunction with common regulatory mechanisms are involved. Furthermore, the DNA bending or changes in local DNA topology/organization also appear to play an important role in the regulation of the expression of phaseolotoxin genes.

### 6.3. Regulation of Phaseolotoxin by OxyR

The regulatory circuitry of the oxidative stress response had already been suggested to be involved in the expression of phaseolotoxin genes (as mentioned above) [38]. The influence of the OxyR global regulator in phaseolotoxin synthesis was demonstrated by the obtaining of a non-toxigenic (Tox-) phenotype in a *P. savastanoi* pv. *phaseolicola oxyR-* mutant strain [43]. Expression analyses showed that the OxyR protein positively influences the expression of the Pht cluster genes at low temperatures (18 °C). The thermoregulation of the genes in the Pht cluster by low temperatures (18 °C) is lost with a lack of the OxyR protein, obtaining no transcript signals at 18 °C for genes representatives of each transcriptional unit (*argK*, *phtA*, *desI*, *phtL*, and *amtA*) that makes up this region. Similarly, the expression dependent on low temperatures (18 °C) of the *pboO*, *pboL*, and *pboA* genes of the Pbo cluster is lost in an *oxyR^−^* mutant background, demonstrating the key role of the OxyR protein in the synthesis and expression of phaseolotoxin genes under low temperature conditions (18 °C) [43]. In addition to its role as a positive regulator of most of the phaseolotoxin genes at low temperatures, the OxyR protein also exerts an influence on the expression at 28 °C of a few of these genes. The constitutive expression of the *pboJ* monocistronic gene at both 28 °C and 18 °C is lost in the absence of the OxyR protein [43]. OxyR, belonging to a family known as the LysR type of DNA-binding proteins has been identified as an oxidative stress sensor in the cells, in particular of the peroxide (H_2_O_2_), and as one of the main regulators for the response to oxidative stress. Although OxyR is primarily thought of as a transcriptional activator, in some bacteria it can function as either a repressor or an activator under both oxidizing and reducing conditions [71,72,73]. The OxyR regulon has been widely studied in the *E. coli* and *P. aeruginosa* bacteria. Although there are significant differences, the OxyR regulons of these and other microorganisms tend to include similar classes of genes, mainly those involved in the defense to oxidative stress and iron homeostasis [72,74,75].

Diverse studies have demonstrated the coordinated regulation of iron metabolism with oxidative stress defenses [76]. The influence of OxyR in iron homeostasis has been widely documented. In *E. coli*, the OxyR protein activates the expression of Fur, the global repressor of ferric ion uptake [77]. In *Rhodobacter sphaeroides*, a regulatory link mediated by *OxyR* has been established between oxidative stress response and iron limitation [78]. Likewise, *OxyR* positively influences the expression of the *pvdS* gene, encoding the ECF sigma factor that is required for the expression of the pyoverdine siderophore biosynthesis genes in *P. aeruginosa*, while the expression of *fur* is not under the control of OxyR in this microorganism [74]. Regarding *P. savastanoi* pv. *phaseolicola* NPS3121, a positive effect of the OxyR protein has been demonstrated on the synthesis of pyoverdine, a major yellow-green Fe(III) chelating siderophore. OxyR influences the production of this siderophore by the positive control it exerts on the expression of the gene encoding the PvdS sigma factor, which is involved in the synthesis of this pigment [43]. Thus, again, the relation of iron metabolism to regulators and conditions (e.g., oxidative stress) related to phaseolotoxin synthesis is evident. This relation could be explained through the positive control that OxyR exerts on the *phtL* gene expression (Pht cluster) essential in phaseolotoxin synthesis (mentioned above), whose product in turn represses the expression of the Fur protein, carriying out the release of the repression exerted on the *pvdS* gene, which favors the expression of iron-uptake genes. However, the PhtL protein only influences the expression of *phtM-phtV* (Pht cluster) and the *pboK-pboN* operon (Pbo cluster), while OxyR favors the expression of all genes of the Pht and Pbo cluster; this suggests that alternative regulatory pathways to PhtL but related to OxyR are involved in the expression of the Pht and Pbo cluster genes.

### 6.4. Regulation by Bacterial Two-Component Systems

Two-component systems (TCSs) are the link between bacteria sensing environmental signals and regulating their physiological behaviors as an adaptation to the environment. The GacS/GacA (global activator of antibiotic and cyanide production) TCS is a global signal transduction system highly conserved in Gram-negative bacteria and prevalent in *Pseudomonas*, which regulates a wide variety of physiological processes including phytotoxins [61]. Sensor kinase GacS responds to yet-to-be-identified environmental signals and activates the cognate response regulator GacA by a phosphorelay mechanism. Once activated, GacA regulates the production of several small untranslated regulatory RNAs (sRNA) (e.g., RsmX, RsmY, and RsmZ), which bind to the repressor proteins, RsmA/E (Csr/Rsm system), and relieve the translational inhibition on target genes. So far, the only known targets of GacA are the genes for the antagonist sRNAs, so it is assumed that the effects of GaS/GacA are mediated mostly via the Csr/Rsm system. However, there is not a complete overlap between the Csr/Rsm and the GacS/GacA regulons [61]. Studies in *P. syringae* have demonstrated that the functionality or regulation mechanisms/targets of GacS/GacA have diversified even at the level of isolates [79]. The obtaining of a non-toxigenic (Tox-; non-producers phaseolotoxin) phenotype in *P. savastanoi* pv. *phaseolicola gacA-* mutant strains (NPS3121 and 1448A strains) and in a *P. syringae* pv. *actinidiae* A18 *gacA-* mutant indicated the participation of the GacS-GacA two-component system in phaseolotoxin synthesis [61,80,81]. By microarray analysis and/or RT-PCR assays, it was demonstrated that the GacS-GacA two-component system regulates the low temperature-dependent (18 °C) expression of most of the Pht cluster genes. The expression of the *phtA*, *phtD*, *phtL*, and *phtM* operons at 18 °C was downregulated in a *P. syringae* pv. *phaseolicola* NPS3121 *gacA-* mutant background, thereby suggesting a positive control for the Pht cluster genes mediated by the GacS-GacA system. Similarly, the positive influence of the GacA response regulator on the expression at low temperatures (18 °C) of the genes *pboN* (PSPPH_4546), *pboA* (PSPPH_4550), *pboE* (PSPPH_4553), *pboF* (PSPPH_4554), and *pboG* (PSPPH_4555) belonging to the Pbo cluster in *P. syringae* pv. *phaseolicola* NPS3121 was demonstrated [80]. The implication of GacA on the expression of the rest of the transcriptional units (*pboO* and *pboJ*) of the Pbo cluster has still not been evaluated.

On the other hand, the implications of the GacS-GacA system on the expression of phaseolotoxin genes, particularly those of the Pht cluster, were observed to not only occur under low temperature conditions. At 28 °C, the overexpression of the *argK* gene in a *P. syringae* pv. *phaseolicola* NPS3121 *gacA-* mutant strain was demonstrated using RT-PCR analyses. The low temperature-mediated (18 °C) thermoregulation for the expression of the *argK* gene was lost with a lack of the GacA protein, showing a constitutive expression at both 28 °C and 18 °C [80]. This might suggest a role for GacA as negative regulator of the *argK* gene at 28 °C. However, so far the experimental evidence does not allow the conclusion that this is a direct role or even rule out the possibility that GacA participates in the negative control of some activator of the *argK* gene at 28 °C. As mentioned above, so far only two additional regulatory pathways related to *argK* gene expression have been identified: the *phtABC* gene products (Pht cluster) that participate indirectly in the repression of the *argK* gene at 28 °C by their action in the synthesis of a corepressor carrying out the activation of an unknown repressor protein [58] and the thermoregulation of *argK* mediated by a repressor at 28 °C which binds to the TRR sites present in the promoter region of this gene [18,60]. Further, there is also the induction mediated by carbamoylphosphate [19]. Thus far, there is no experimental evidence that allows a link to be established between one or both of these regulatory mechanisms and that mediated by the GacS/GacS two-component system. Likewise, there is no evidence relative to the influence of GacS/GacA on the expression of the rest of the Pht cluster genes at 28 °C.

The way by which the GacS/GacA system regulates the synthesis and phaseolotoxin genes (Pht and Pbo cluster) is poorly understood. Some efforts aimed at elucidating molecular components of the signal transduction pathway GacS/GacA have demonstrated the influence of the *rsm* system in phaseolotoxin synthesis in *P. syringae* pv. *phaseoliocola* NPS3121. The overexpression of the *rsmA* gene of *P. aeruginosa* PAO1 in the *P. syringae* pv. *phaseoliocola* NPS3121 strain by using a low-copy-number plasmid (pSK61) produced a substantial reduction in phaseolotoxin production [82].

Recently, advances in the knowledge of the signaling pathway of the GacS/GacA system related to phaseolotoxin synthesis have been made. Using bioinformatics analyses, seven *rsm* gene homologues were identified in the *P. amygdali* pv. *phaseolicola* 1448A genome. The genes encoding RsmA, RmsC, RsmE, and RsmH1 are located in the chromosome while the other three, *rsmH2*, and two identical copies of *rsmH3* (*rsmH3-1* and *rsmH3-2*), are found in the virulence plasmid p1448A-A [61]. The RNAseq analyses of the *P. amygdali* pv. *phaseolicola* 1448A strain grown at 28 °C and 18 °C showed that most of the *rsm* genes did not show changes in their expression levels at 18 °C or 28 °C, with the exception of *rsmE*, which was significantly overexpressed at 18 °C. Similarly, the *gacA* transcription is not regulated by temperature. Furthermore, the transcriptomic analyses by the RNAseq of a *P. amygdali* pv. *phaseolicola* 1448A *gacA-* mutant strain grown at 18 °C or 28 °C showed that only the *rsmE* and *rsmH3* genes are dependent on the GacS/GacA system, whose effect is a function of the temperature. The expression of *rsmE* at 18 °C was reduced in the *gacA-* mutant background indicating that GacA acts as an activator of *rsmE* at 18 °C. The expression of *rsmH3-1* and *rsmH3-2* was induced at 28 °C in the *gacA-* mutant, suggesting a negative control mediated by GacA in the expression of these genes at 28 °C [61]. Additionally, the RNAseq transcriptomic analyses of the seven regulatory small RNAs (*rsmX1-5*, *rsmY* and *rsmZ*) suggests a main role in gene regulation for the *rsmX1*, *rsmX2*, *rsmX3*, and *rsmY* genes on the basis of their higher expression levels in relation to the other sRNA antagonist genes at both 28 °C and 18 °C. Only four of these sRNA genes (*rmsX1*, *rsmX4*, *rsmX5*, and *rsmZ)* showed lower levels of expression at 18 °C compared to 28 °C, suggesting that they have a different role in the thermoregulation of gene expression in *P. amygdali* pv. *phaseolicola* 1448A [61]. The expression of the seven sRNA antagonists is dependent on the GacS/GacA system, since lower expression levels in these genes are obtained in a *gacA-* mutant background at both 18 °C and 28 °C, with the exception of *rsmZ*, whose expression at 28 °C was not affected by the loss of the GacA protein but whose expression at 18 °C was. These results suggest a positive role of the GacS/GacA system on the expression of the sRNA antagonist genes in *P. amygdali* pv. *phaseolicola* 1448A [61]. Furthermore, these analyses showed that GacA stimulates its own transcription at 28 °C.

By constructing diverse combinations of single, double, and multiple mutations for the *rsm* genes in *P. amygdali* pv. *phaseolicola* 1448A, and evaluating the virulence capacity in these mutants strains, it was shown that only the *rmsA- rsmE-* mutant strain was impaired in its virulence capacity in bean plants and in phaseolotoxin synthesis at 18 °C, suggesting that only these two genes are required redundantly for full virulence. These results suggest a positive role for RsmA and RsmE in phaseolotoxin synthesis at 18 °C [61].

The effect of RsmA and RsmE in phaseolotoxin synthesis is dependent on the growth stage because the marked reduction in the production of this antimetabolite was observed only in cultures in early stages of growth of the *rmsA- rsmE-* mutant strain while cultures in the stationary phase produced inhibition haloes similar to those produced by the wt strain. A slight decrease in the phaseolotoxin production was observed in the single *rsmA*- mutant at 18 °C, whose effect was only observed in early stage growth cultures [61].

Surprisingly, the analyses in 1448A mutant strains overexpressing the different *rsm* homologues showed contradictory results to those mentioned above. The *rsmE* overexpression suppressed the production of phaseolotoxin at 18 °C, particularly at early stages of growth (14–21 h), and this repression was partially alleviated in older cultures (approximately 48 h). In turn, *rsmC*, *rsmH1*, or *rsmH2* overexpression lead to a small decrease in the production of phaseolotoxin at both growth stages in relation to the wt strain. The *rsmA* overexpressing mutant showed only a slight decrease in the phaseolotoxin synthesis in exponential growth cultures. No phaseolotoxin synthesis was observed at 28 °C in either mutant or overexpressed mutant strains [61]. These opposing phenotypes observed from the overexpression of the *rsm* genes were suggested to be artefactual because of an abnormally high concentration of the regulator leading to possible non-specific interactions or the destabilization of the relative concentrations of different molecules involved in regulation, such as sRNAs [61]. Based on the global expression patterns observed by RNAseq and mutants strains analyses in the components of the GacS/GacA and Rsm systems, the existence of a regulatory molecule different from the Rsm system but dependent on the GacS/GacA system had been suggested to be involved in the repression of phaseolotoxin synthesis at 28 °C [61].

For now, the relation between the GacS/GacA TCS with those regulatory protein previously identified as involved in the expression of phaseolotoxin genes such as the IHF protein (*phtD* promoter binding) [51] and the putative 14–20 kDa regulatory protein of the *phtM* operon (mentioned below) [70] could be ruled out since the synthesis and binding ability to the respective promoter regions of these proteins (IHF and 14–20 KDa putative regulatory protein) are independent of the GacS/GacA two-component system [51,70].

### 6.5. Unidentified Putative Regulatory Proteins 

The participation of a 14–20 kDa DNA-binding protein in the regulation of the *phtM-phtV* operon (Pht cluster) has been postulated. The identity of this protein has not been determined; however, the studies performed with other pv. *phaseolicola* strains (e.g., CLY233; non toxigenic; non-carrier of Pht cluster) and with other *P. syringae* pathovars (non-phaseolotoxin producers) revealed that this 14–20 kDa protein corresponds to a global regulator [70]. The binding site of this 14–20 kDa putative regulatory protein was delimited to a 58 pb region spanning the −43 to +14 positions relative to the transcription initiation site of *phtM*, which appears to be exclusive to toxigenic (phaseolotoxin producers) *P. syringae* strains because its presence is conserved in only these strains. Although target sequences for transcription factors have not been identified in this 58 pb region by in silico analysis, a repressor function for this 14–20 kDa DNA-binding protein has been proposed on the basis of the position of the binding site, which is characteristic of repressor proteins [70,83]. This 58 bp region still contains four of the six conserved regions between the *phtD* and *phtM* operons (mentioned above), which were postulated as targets of a common regulatory pathway among both operons [14]. However, a 14–20 kDa putative regulatory protein was demonstrated that does not correspond to the common regulation mechanism suggested. The 14–20 kDa putative regulatory protein is specific to *phtM*, since it has only the ability to bind to this region and not to the *phtD* upstream region [70]. More experimental work is necessary to evaluate the binding ability of this 14–20 kDa putative regulatory protein on the rest of the promoter regions of the Pht cluster genes, even in those belonging to the Pbo cluster. The latter is due to the implications of certain regulatory mechanisms on the gene expression of both clusters (e.g., PhtL protein). Through molecular mass (MM) fractionation and gel shift assays, the functioning of the 14–20 kDa putative regulatory protein was characterized as a monomer or multimer of the same protein in its binding to the *phtM* promoter. Furthermore, the studies evaluating the link between this protein and regulatory mechanisms identified as also involved in *phtM* operon expression, particularly the GacS-GacA system, demonstrated that this regulatory pathway that involves the 14–20 kDa protein appears to be independent of the GacS-GacA system because its production and *phtM* promoter binding is not affected by a lack of the GacA regulator response [70]. Thus, the current experimental evidence demonstrates that the regulatory pathway of the *phtM* operon mediated by the 14–20 kDa putative regulatory protein corresponds to an alternative pathway to the GacS-GacA TCS and those mediated by the PhtL protein.

## 7. Conclusions and Future Directions

**Figure 4:**
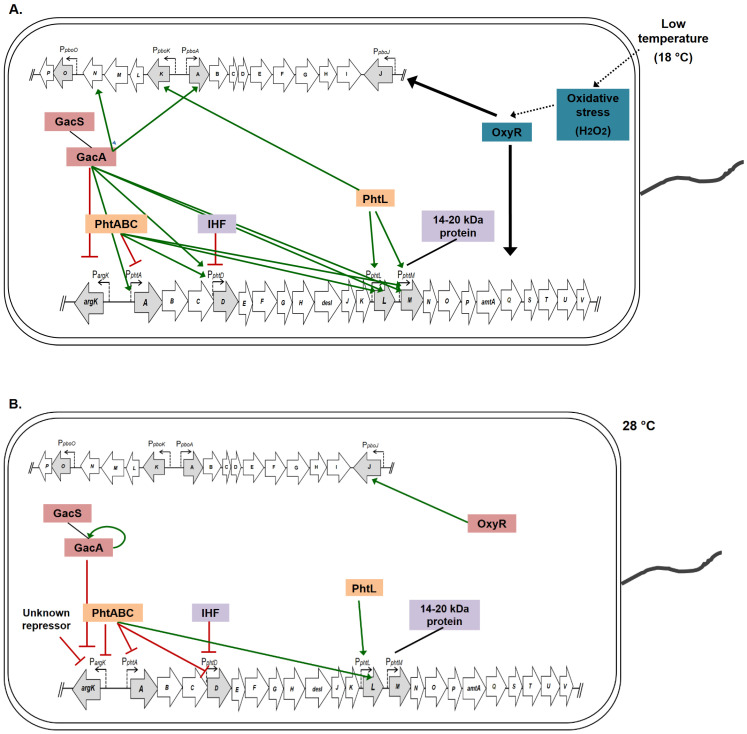
Regulatory proteins involved in the phaseolotoxin genes expression. In the image are integrated the regulatory pathways that influence the expression of phaseolotoxin genes (Pht and Pbo cluster). (**A**) Regulatory pathways involved in the expression at 18 °C of genes related to phaseolotoxin production. (**B**) Regulatory pathways involved in the expression at 28 °C of genes related to phaseolotoxin production. Green arrows mean positive control and the red interrupted lines means negative control. The black arrows and blue legend boxes indicate regulators with influence in all genes of the Pht and Pbo clusters. Pink legend boxes indicate proteins with partial influence in phaseolotoxin genes while orange boxes indicate regulators encoded in the Pht cluster. The purple boxes (IHF and 14–20 kDa protein) indicate proteins whose function, as influenced by temperature, has not been demonstrated.

Current advances in the regulatory mechanisms and/or signal transduction pathways involved in the expression of phaseolotoxin genes highlight the complexity of this process, in which regulation mechanisms at the transcriptional and posttranscriptional level are involved, mediated by both specific regulatory proteins and global regulators (Table 2 and Figure 4). Some of these have dual roles in the control of various genetic determinants of phaseolotoxin and the function of the sensed signals. Furthermore, changes in the topology or DNA structural organization appear to also contribute to the fine control of the genetic traits related to phaseolotoxin production. From the integration of the regulatory elements and signals related to phaseolotoxin synthesis, it is now possible to strengthen the proposals for the participation of certain elements that were seen as involved in the process. In this sense, the relationship of iron with different regulatory molecular elements of phaseolotoxin places it as a potential element belonging to the signaling pathway related to the synthesis of this compound. The influence of this element (iron) on the synthesis and expression of phaseolotoxin genes and related regulatory proteins is currently being evaluated in our working group. Likewise, the integral analysis established in this work allow us to glimpse mechanisms and processes that could be related to the expression of phaseolotoxin genes. The knowledge we have gained on phaseolotoxin regulation is significant and continued work in this area will reveal more about the evolutionary and adaptive strategies of phaseolotoxin producers on specific regulatory factors, and shed more light on the relationship between virulence and physiological cellular context.

## Figures and Tables

**Table 1:** Signals and environmental parameters with influence on phaseolotoxin synthesis.

Signal/Parameter	Conditions	Effect(s)	Reference
Low temperature	Bacterial cultures, 8 °C to 18 °C	High levels of phaseolotoxin production in cultures	[32]
		Overexpression of most of the phaseolotoxin genes (Pht and Pbo clusters)	[14]
		Intracellular oxidative stress	[33]
Incubation Time	Bacterial cultures, first 24 h In host plant, 2–3 days post-inoculation	Production of phaseolotoxin is performed	[34]
Medium/Carbon source	Sucrose	Greater phaseolotoxin production	[35]
	HS and HSC media	Induction of phaseolotoxin genes (*argK*, and *argD*)	[36]
Plant metabolites	Bean leaf extracts and apoplastic fluidPod extracts	Enhance the expression of most Pht cluster genes at 18 °C	[37]
		Diminished expression of the Pbo cluster genes	[37]
Oxidative stress	1 mM H_2_O_2_	Expression at 28 °C of the Pht cluster genes	[38]
	Superoxide ion (O_2_^−^)	Positive and negative effects on the expression of the Pht cluster genes at 28 °C	[38]
Phaseolotoxin precursors	Carbamoyl-phosphate	Induce the expression of the *argK* gene at 28 °C	[19]

**Table 2:** Summary of regulatory elements involved in phaseolotoxin synthesis.

Regulatory Element	Condition	Effect(s)	Reference
**Positive effect**			
*Cis* element			
Thermoregulatory region (TRR)	28 °C	Titrates to unknown repressor molecule leading to phaseolotoxin production	[59]
Regulatory protein			
OxyR	18 °C	Influences on the expression of genes of the Pht and Pbo clusters	[43]
GacS-GacA system	18 °C	Positive control for most of the genes of the Pht and Pbo clusters	[80]
Regulatory RNA			
Rsm system (RsmaA and RsmE)	18 °C	Positive role for RsmA and RsmE in phaseolotoxin synthesis	[61]
**Negative effect**			
Regulatory protein			
Unknown repressor	28 °C	Inhibition of phaseolotoxin synthesis	[18]
IHF	Unidentified	Partial repression of *phtD-phtK* operon (Pht cluster)	[51]
GacS-GacA system	28 °C	Negative control of *argK* gene	[80]
Putative regulatory protein 14–20 kDa	Unidentified	Putative repressor function of *phtM* operon (Pht cluster)	[70]
